# Pigment cell movement is not required for generation of Turing patterns in zebrafish skin

**DOI:** 10.1038/ncomms7971

**Published:** 2015-05-11

**Authors:** D. Bullara, Y. De Decker

**Affiliations:** 1 Département de Chimie, Center for Nonlinear Phenomena and Complex Systems (CENOLI), and Nonlinear Physical Chemistry Unit, Université libre de Bruxelles (ULB), Campus Plaine, C.P. 231, Brussels 1050, Belgium

## Abstract

The zebrafish is a model organism for pattern formation in vertebrates. Understanding what drives the formation of its coloured skin motifs could reveal pivotal to comprehend the mechanisms behind morphogenesis. The motifs look and behave like reaction–diffusion Turing patterns, but the nature of the underlying physico-chemical processes is very different, and the origin of the patterns is still unclear. Here we propose a minimal model for such pattern formation based on a regulatory mechanism deduced from experimental observations. This model is able to produce patterns with intrinsic wavelength, closely resembling the experimental ones. We mathematically prove that their origin is a Turing bifurcation occurring despite the absence of cell motion, through an effect that we call differential growth. This mechanism is qualitatively different from the reaction–diffusion originally proposed by Turing, although they both generate the short-range activation and the long-range inhibition required to form Turing patterns.

## Abstract (web summary)

Pigment pattern formation in zebrafish depends on the interactions between the pigment cells. Here the authors present a mathematical model based on mutual interaction of pigment cells in the absence of cell motion, revising the current model of the pattern formation that relies on reaction–diffusion Turing patterns.

## Graphical abstract

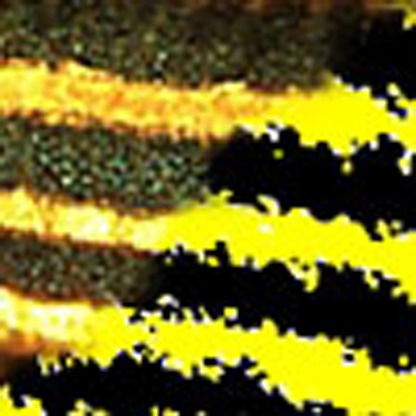

In recent years, developmental biology experienced a renewed interest in one of its most fascinating and long-debated phenomena: morphogenesis^1^. The emblematic question of morphogenesis is how the egg cell, which is essentially a round supramolecular aggregate, can spontaneously break its original symmetry to produce the very complex shapes of the adult body, in such a diverse yet precise and reproducible way.

A hypothesis for such a mechanism was proposed by Alan Turing in 1952 in his seminal paper^2^. Turing shows with a theoretical model that a chemical reaction coupled with Fickian diffusion can give rise to a spontaneous symmetry-breaking phenomenon, in which an initial state having a uniform distribution of chemicals is converted into a regular pattern of concentrations. He formulated his ideas in the framework of the reaction–diffusion (RD) formalism, with a mechanism that can be shortly explained as follows. A chemical species X locally promotes its own production because it is part of an autocatalytic reaction. This chemical feedback results in a short-range activation of X. The reaction also creates a species Y that promotes the consumption of X. If Y diffuses more rapidly than X, this second chemical feedback leads to a long-range inhibition. The combination of these two effects induces a non-trivial spatial distribution of the concentrations, which generates the patterns. Since this pioneering work, the literature has referred to such mechanism and to the corresponding stationary patterns, respectively, as Turing instability and Turing patterns.

Turing's hypothesis is fascinating because it reduces the enormous complexity of the original biological problem to a relatively ‘simple' chemical explanation. Thanks to this simplicity, chemical Turing patterns have been extensively investigated for decades, both theoretically^3^ and experimentally^4^,^5^. The basic thermodynamical conditions for their emergence has been clarified by Prigogine *et al*.^6^,^7^. It is now well known that Turing patterns are a typical example of dissipative structures. These structures are a class of spatiotemporal organizations that can be obtained only far from equilibrium, where a system can continuously dissipate entropy in its environment, which compensates the entropy produced by the internal irreversible processes and keeps the system in an organized state.

From a mathematical perspective, a Turing mechanism is a mechanism that generates a Turing bifurcation (the ‘Turing bifurcation' is more often called ‘Turing instability' in the literature. To avoid any confusion, we use the term ‘bifurcation' when referring to the mathematical condition, and ‘instability' in a more general fashion). A Turing bifurcation can occur in the system of partial differential equations (PDEs) ruling the spatiotemporal evolution of a set of dynamical variables **x**={*x*_*i*_(**r**, *t*)}


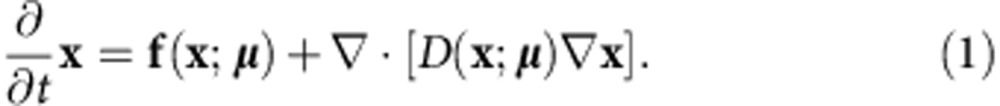


In these equations, **f** stands for the local evolution laws, which are usually nonlinear and depend on a set of control parameters ***μ***. The second term accounts for the diffusion of the different variables based on the matrix of diffusion coefficients *D*(**x**;***μ***). The bifurcation takes place when one of the eigenvalues *ω*(*k*) of the corresponding linear problem is real and crosses zero for a unique and nonzero wavenumber *k*=*k*_T_. This condition, which is controlled by the value of the parameters, implies that the dominant unstable mode of the bifurcating solution has an intrinsic wavelength 2*π*/*k*_T_.

To understand the detailed mechanism underlying pattern formation in vertebrates, the zebrafish *Danio rerio* has been proposed more than 10 years ago as a model organism for experimental investigations^8^. The reasons for this choice are, on the one side, the ease and rapidity of breeding and genetically studying large colonies of these small animals, and on the other side, the large variety of coloured skin patterns that can be observed in different mutations within the Danio genus. These patterns range from stripes to spots of different sizes, and strikingly resemble the family of Turing patterns which are produced by classical RD models^9^. This analogy further extends to the dynamical response that both zebrafish skin patterns and theoretical Turing patterns have with respect to perturbations^10^.

These findings might suggest that a RD mechanism underlies the formation of the zebrafish's skin patterns, but there are several points against this hypothesis. The first is that the pattern is not formed by a continuous concentration field of pigment molecules diffusing across the fish skin, but by a discrete assembly of coloured cells—melanophores (black), xantophores (yellow) and iridophores (light blue)—whose motion is unlikely to follow a Fickian dynamics. The second is that RD systems are not the only ones that can create patterns with an intrinsic wavelength in biology, as evidenced by Höfer and Maini^11^ for the angelfish^12^. Moreover, the size of the pattern is very small, with a characteristic wavelength of only 10–20 cells (around 1∼2 mm). Such numbers endanger the straightforward application of spatially continuous models. So, clearly we are not in the presence of a classical RD system, yet the analogies with RD models cannot be disregarded as mere coincidences.

To gain more insight on the underlying mechanisms, Kondo *et al*. performed controlled experiments to identify the mutual interactions between xantophores and melanophores. These studies unveiled a regulatory network in which the two types of cells inhibit each other at short range, while xantophores activate the emergence of melanophores at long distances^13^. Remarkably, the proposed mechanism does not seem to require diffusion nor any kind of cell motion. This hypothesis has been recently consolidated by experimental investigations by Nüsslein-Volhard *et al*. These authors showed that the Kondo mechanism is a subset of a more complex regulatory scheme involving iridophores^14^. More importantly, the authors also stress that skin patterns emerge despite the absence of extensive cell movement^15^.

All these studies suggest that patterns can emerge in the case of immobile units. To test whether this hypothesis is correct, we derive and analyse in this work a mathematical model for immobile cells based on the observed regulatory network. We show that this model can undergo a Turing bifurcation and reproduce the experimental patterns. We provide in this way a proof of principle for the idea that Turing patterns can appear in systems made of immobile agents.

## Results

### Rationale for the choice of model

As mentioned above, the investigations by Kondo *et al*. led to the conclusion that melanophores and xantophores regulate each other's growth. Nüsslein-Volhard *et al*. arrived at the conclusion that iridophores also play a role in the emergence of the skin patterns. They observed that patterns can be seen in fish mutants having at least two of the three types of cells. They thus proposed that on top of the mechanism put forth by Kondo, interactions also exist between melanophores and iridophores, which are qualitatively the same as the ones between melanophores and xantophores. Since xantophores and iridophores also regulate each other's growth, the resulting system actually consists in two coupled Kondo-like mechanisms.

Our objective is to assess with a theoretical model whether immobile cells presenting short-range activation and long-range inhibition can lead, generally speaking, to patterns that are similar to those observed in the experiments. We also want to understand the mechanism by which such patterns would emerge. Despite the tremendous amount of experimental work, such a model does not exist yet. Even the complicate multiscale model proposed by Nakamasu *et al*. in ref. 13 implicitly relies on diffusion. Consequently, we devise a scheme that is solely based on the interactions between xantophores and melanophores.

This choice is also justified by recent studies^16^ showing that iridophores are indeed necessary to develop patterns in the trunk of the fish, but that melanophores and xantophores alone are enough to produce the fin patterning. Since the pattern in the trunk is contiguous with the one in the fins, and since the two share the same geometrical characteristics, it is reasonable to think that they should also share the same core mechanism. In other words, melanophores and xantophores are central to pattern formation, with iridophores playing mainly an assistance role in the trunk.

A classical route to model morphogen gradients is to experimentally individuate what kind of transport mechanism the morphogens are subject to, and write a set of PDEs for their concentration field variables accordingly^17^. In the case of diffusive motion, one obtains a set of equations of the form (1). This implies the presence of morphogens undergoing a simple Brownian motion^18^, which is not the case here. To cope with more complex situations, different approaches exist in the literature, involving, for example, integro-differential diffusion^19^,^20^ or statistical mechanics modelling^21^.

In the present case, the patterns are formed by a discrete assembly of coloured cells interacting with each other, and we have experimental information on the network of such cell-to-cell interactions: it seems therefore a natural choice to describe the fish skin at its cellular level, as a discrete system. This will allow us to translate the cellular interactions into a set of probabilistic processes, from which average evolution equations can be derived. This approach has been successfully applied at the level of chemical reactions taking place on low-dimensional supports^22^. Its generality makes it suitable as well for the kind of cellular model we will use.

### Model derivation

To simplify the mathematical setting, we describe the fish hypodermis as a regular lattice, whose nodes can either be occupied by a xantophore, by a melanophore or by another type of cell. We thus exclude the possibility of overlapping between the two types of chromatophores. This approximation is supported by the experimental evidence that melanophores and xantophores rest on separate layers of iridophores^23^. The distance *a* between two nodes is chosen to be the average diameter of a chromatophore. The node at position *i* in the lattice is described by three boolean variables, *X*_*i*_ for the xantophores, *M*_*i*_ for the melanophores and *S*_*i*_ for a node without chromatophore, which take the value 1 if the node is occupied by the corresponding species, and 0 otherwise. Consequently, one has *X*_*i*_+*M*_*i*_+*S*_*i*_=1 everywhere and at all times.

The melanophores can undergo different types of transformations and interactions. We choose to represent these different events as stochastic processes, each having an intrinsic probability per unit time. The change in the cellular nature of each node is thus accounted for by a modification of the local variables, induced by each of the processes. We will now identify these events, and see how they translate in the framework of the probabilistic description that we propose.

The precursors of the chromatophores develop in the neural crest of the fish, and then migrate towards the skin through essentially two pathways: a ventromedial pathway between the neural tube and somites, and a dorsolateral pathway between the somites and the epidermis^8^,^24^. It is also known that the two pathways are not equally selected by neural crest cells, especially in the early stage of embryonic development, and that this may cause the establishment of a pre-pattern on the fish skin, which helps to determine the fine details and the orientation of the final pattern. Singh *et al*.^25^ showed that the development of the pre-pattern and the final pattern in the growing fish is strongly influenced by the presence of iridophores alongside melanophores and xantophores.

However, we will not include these details in our description. Our objective is to develop a two-species model that can assess whether the regulation scheme that is at the heart of the qualitative mechanism proposed by Kondo is able to generate patterns with an intrinsic wavelength and geometry. We will thus simply consider that, thanks to the above two pathways, chromatophores can randomly appear at any position in the hypodermis that is not already occupied by a chromatophore. We call this process the ‘birth' of either a xantophore or a melanophore. In symbolic notation, the birth processes read


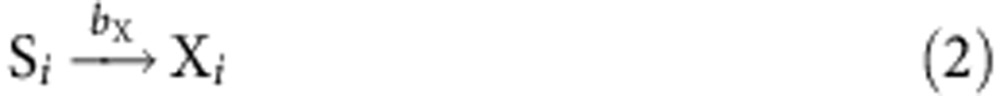



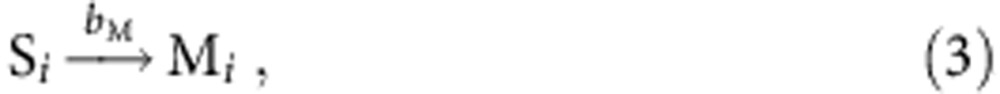


where *b*_X_ and *b*_M_ are the rate constants characterizing the probability for a xantophore or a melanophore to appear, per unit time, at a node *i*. We denote the nature of the cell with straight letters corresponding to the boolean occupation variables introduced earlier.

Chromatophores can also naturally die because of ageing processes. When a chromatophore dies it is rapidly destroyed and removed from the hypodermis, leaving room for a new chromatophore. We write these two natural death processes as:


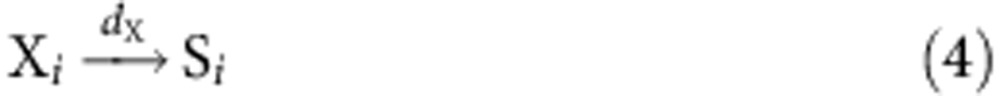



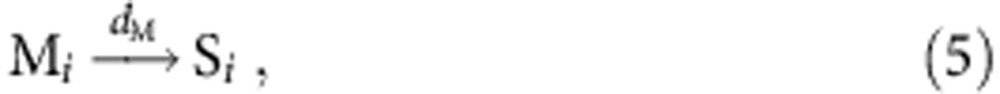


where *d*_X_ and *d*_M_ are the corresponding rate constants.

Experiments performed *in vivo* in the early stage of pattern development show that xantophores and melanophores mutually inhibit each other's birth when they are close^13^. More precisely, the experiments show both an increase in the proliferation of one type of cell when the first neighbouring cells of the other type are ablated, and an increased death rate of one type of cell when it is surrounded by cells of the other type. These observations suggest that such short-range interaction is mediated either by direct contact between the two cells^26^, or by local competition for nutrients. In either case, it is safe to assume that the range of this mutual inhibition extends only to first neighbours. We will consequently cast the overall effect of this competition into an increased death probability of a xantophore (melanophore) when a melanophore (xantophore) is close-by:


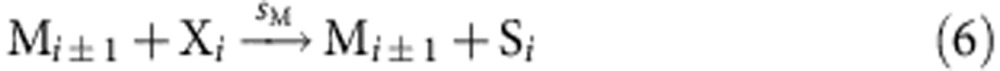



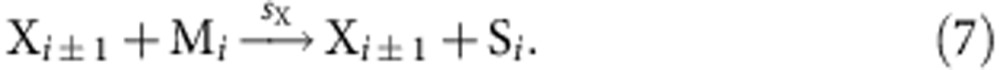


The rate constants *s*_M_ and *s*_X_ are assumed to be larger than their natural counterparts *d*_X_ and *d*_M_.

Chromatophores can influence each other's growth also when they are separated by a few cells^13^. We will refer to this kind of feedback as long-range interactions. These experiments have been performed on striped skin patterns, by selectively ablating cells belonging to a certain stripe while monitoring the birth or death rates of a group of cells in a neighbouring stripe. Therefore, we infer that such long-range interactions extend over a characteristic distance, which we call *h*. It would appear from the experiments that *h* is typically of the order of 10 cells, which corresponds to one half of the wavelength of the stripes. We would like to stress that at this stage, we do not relate *h a priori* to this wavelength. This parameter simply acknowledges the fact that there must be a long-range interaction between a cell at position *i* and another one at position *i*±*h*. If the model is representative of the experiments, we should find *a posteriori* that the wavelength of the pattern is approximately equal to 2 *h*.

Three types of long-range interactions have been experimentally observed:

- the birth of new melanophores is promoted by the presence of xantophores at a distance *h*;
- the survival of already existing melanophores is enhanced by the presence of xantophores at a distance *h*;
- the birth of new melanophores is inhibited by the presence of already existing melanophores at a distance *h*.

Our investigations of different theoretical models revealed that the process (a) is mandatory to obtain patterns with a wavelength that is controlled by *h*. Processes (b) and (c) bring small modifications to the dynamics but do not affect the patterns qualitatively. Therefore, in accordance with our aim of providing a minimal model, we will take into account only the increased birth rate of melanophores due to the presence of xantophores at long distance:


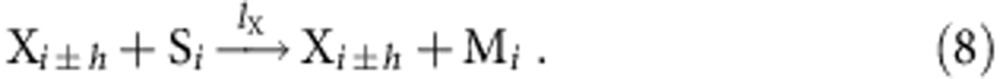


Although the study by Nakamasu *et al*.^13^ shows that a feedback of the type (a) must exist at a distance *h*, it does not say anything about its physico-chemical nature. A recent study by Hamada *et al*. strongly suggests that feedback (b) is mediated by a Delta-Notch signalling occurring at the tip of a long-range projection extended by melanophores, over a distance of approximately one stripe^27^. A similar interaction might occur between already emerged xantophores and melanophores that are below the hypodermis, which would then cause feedback (a), but there is no experimental study so far that confirms or rules out this hypothesis. Such details are, however, not needed in the simple, phenomenological description that we put forward for the long-range interactions.

Finally, we want to discuss in more detail the validity of our hypothesis on the absence of cell motion. Experiments show that melanophores perform some motion across the fish skin^28^. More recently, a complex motion of melanophores and xantophores was observed *in vitro* and was seen to be triggered by their mutual interaction^29^. In both cases, melanophores tend to get away from the xantophores. Nakamasu *et al*.^13^ also report that some melanophores migrate out of the monitored region both in the test and in the control experiments.

Even though we are aware of a certain degree of mobility of the melanophores, we will consider cells to remain immobile in the present study. This hypothesis can be justified as follows. First, there is no systematic study (to the best of our knowledge) that proves that xantophores are capable of appreciable motion *in vivo*. In fact, the most recent experimental studies point towards a total absence of large-scale cell movement^15^. Second, there is no evidence that this motion is necessary for pattern formation: the fact that there is a movement of cells does not mean that this motion is responsible for the pattern formation itself. Moreover, the Kondo mechanism implies that pattern formation does not require any sort of transport. As our main aim here is to test the validity of this hypothesis, we will neglect any type of cell motion whatsoever. The approach we use could, however, easily be extended to include such effects.

### Evolution equations

The above processes form a set of events, each taking place with a given probability. To investigate the dynamics generated by these processes, one can either perform stochastic (kinetic Monte Carlo (MC)) simulations or analyse the evolution equations for the averages of the boolean variables *X*_*i*_, *M*_*i*_ and *S*_*i*_ over an ensemble of realizations. These equations are obtained from the master equation ruling the evolution of the underlying probability distribution. Consider as an illustration the case of a one-dimensional system. Including all the above events, one obtains:


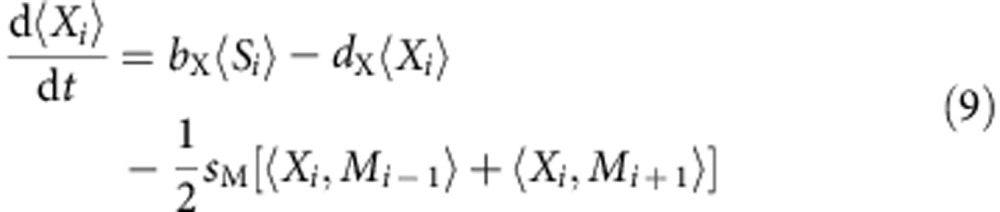



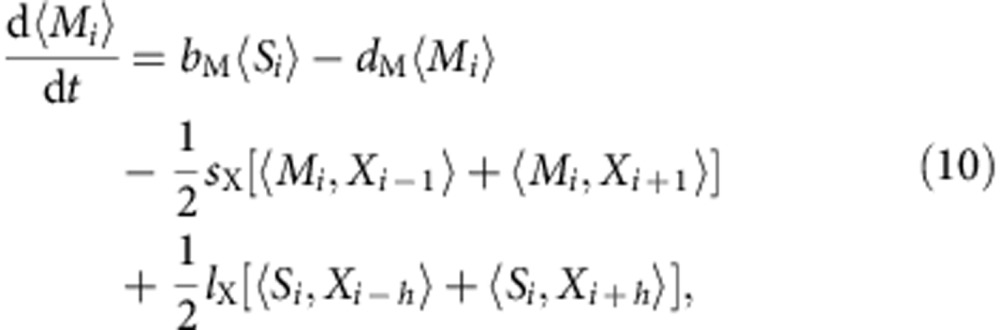


where the brackets stand for ensemble averages. The above equations formally apply to one-dimensional lattices, but can easily be extended to two-dimensional cases. These kinetic laws represent the evolution of the probability for each site *i* to be either occupied by a xantophore or by a melanophore. Note that we do not need an explicit evolution law for 〈*S*_*i*_〉, because of the conservation rule mentioned earlier. The terms of the form 〈*A*_*i*_, *B*_*j*_〉 represent the joint probability of having a particle *A* at position *i* and a particle *B* at position *j*.

These evolution equations can be transformed into relatively simple PDEs in an appropriate limit. First, we use the mean-field hypothesis, which assumes that the average composition of the *i*th node does not depend on the composition of the *j*th node, so that 〈*A*_*i*_, *B*_*j*_〉≃〈*A*_*i*_〉〈*B*_*j*_〉. Second, we switch to continuous spatial coordinates *r*=*ia*, in which *a* is the typical size of a cell. We will denote the corresponding scalar field of average occupation numbers *x*(*r*)=〈*X*_*i*_〉 and *m*(*r*)=〈*M*_*i*_〉, respectively. Finally, we assume that the variations of these newly introduced variables are smooth enough in space so that


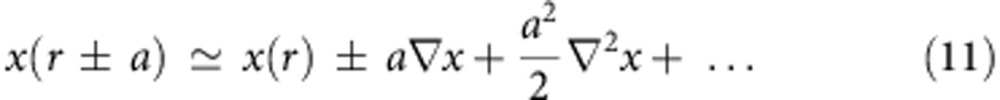



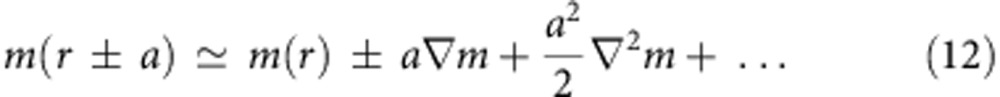



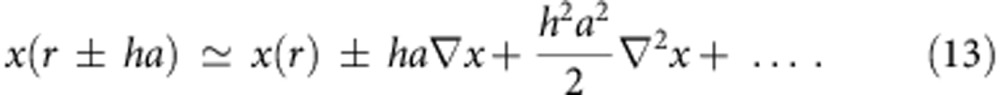


Keeping terms up to the second order in *a*, which acts as an intrinsically small parameter, we obtain the following set of PDEs


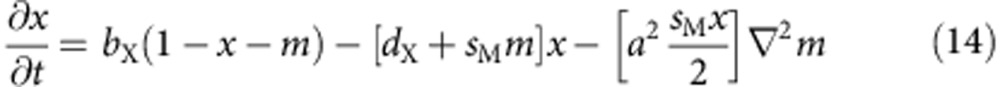



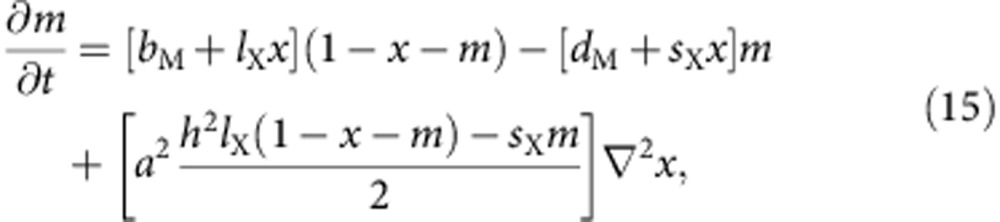


in which we do not explicitly write the spatiotemporal dependences for simplicity. The short-range and long-range interactions translate, in this continuous limit, into cross-diffusion-like terms. These contributions should, however, not be interpreted as being due to cross-diffusion, as their physical origin is completely different: they do not originate from molecular or cell motion, but are instead due to the nonlocal character of the biological interactions between the cells.

We now present the results of stochastic MC simulations of the model, and show how they can be interpreted analytically in terms of the above continuous limit.

### Stochastic simulations

We want to assess whether the proposed model is able to generate stationary structures similar to those observed in the experiments. To this end, we perform simulations of the discrete scheme (2)–(8) on two-dimensional square lattices with relevant values of the parameters.

The experiments show that the short-range inhibition causes cells to die much faster than they would do otherwise. Similarly, the birth of new melanophores is much more rapid when it is promoted by long-range activation by xantophores. For these reasons, we approximate *d*_X_, *d*_M_ and *b*_M_ to zero to obtain a qualitative representation of the behaviour of the system during timescales that are consistent with those of the experiments. In qualitative accordance with the observations, we also assume that melanophores and xantophores inhibit each other with essentially the same strength, so that *s*_X_=*s*_M_=*s*.

**Figure 1:**
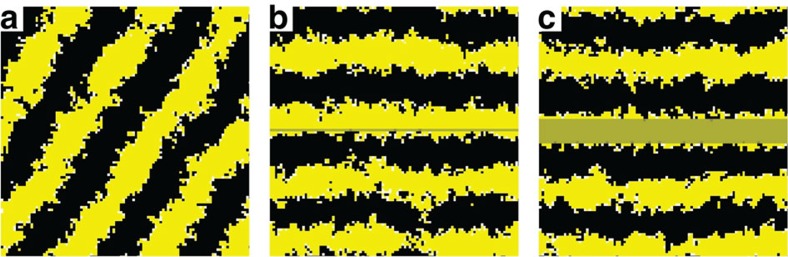
Stationary striped patterns observed in MC simulations. The simulations are performed with a 100 × 100 square lattice with periodic boundary conditions and *b*_M_=*d*_X_=*d*_M_=0, *b*_X_=*s*_X_=*s*_M_=1, *l*_X_=2.5 and *h*=16. They ran for 1 × 10^9^ Monte Carlo steps, starting from an uniform initial condition without xantophores and melanophores. Yellow, black and white boxes represent X, M and S. (**a**) The pattern formation evolves freely. (**b**,**c**) We simulated the presence of an initial horizontal band of iridophores, which inhibit the growth of melanophores on top of them. The iridophores appear as a shaded band, the size of which is 1 cell (**b**) and 10 cells (**c**) wide.

Simulations with such sets of parameters show that the model is indeed able to qualitatively reproduce the experimental patterns (see, for example, Fig. 1).

**Figure 2:**
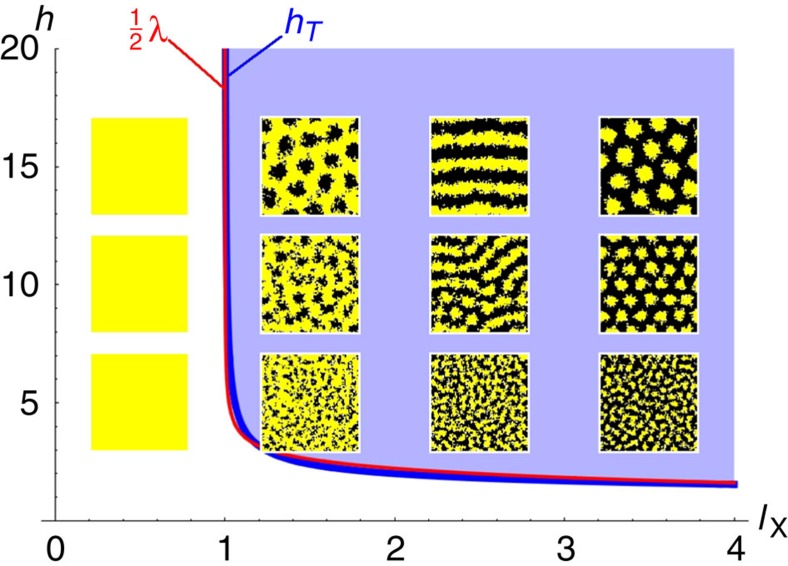
Comparison between the mean-field analytical bifurcation diagram and the MC simulations. The values of the parameters are *b*_M_=*d*_X_=*d*_M_=0, *b*_X_=*s*_X_=*s*_M_=1, *h*={5,10,15} and *l*_X_={0.5,1.5,2.5,3.5}. The blue curve marks the critical values *h*_T_, while the red curve is half of the critical wavelength *λ*.

These patterns are stationary and come in different geometries and wavelengths, which are controlled by the different constants of the model. In particular, if the values of all the other control parameters are fixed, *l*_X_ controls the geometry of the pattern (see Fig. 2). For low values of *l*_X_, the skin would be covered only by xantophores, and would be entirely yellow. Increasing this parameter first turns this homogeneous state into a black-dotted yellow skin, then into a striped pattern and, eventually, into a predominantly black skin with yellow spots. We notice that *l*_X_ barely affects the wavelength of the patterns. It is actually *h* that controls the intrinsic size of the pattern. Remarkably, the wavelength of the pattern turns out to be roughly equal to 2 *h*, as reported in the experimental investigations.

The above results reveal a surprisingly simple selection mechanism for the patterns, which are essentially controlled by the parameters characterizing the long-range interaction: its spatial extent *h* and its ‘strength' *l*_X_. This behaviour can be understood as follows. The mutual short-range competition processes (6)–(7) act as a source of segregation, since they tend to destroy close-by pairs of different chromatophores. Should the birth of chromatophores be controlled only by the random events (2) and (3), one would observe a separation into X and M domains of indefinite size. The only reason why a finite wavelength emerges in our simulations is because the birth of new melanophores is controlled by the long-range interaction. The parameter *h* sets the boundary of a ‘forbidden' region surrounding a xantophore, in which melanophores cannot grow. If *l*_X_ is small, the rate of creation of melanophores is very low and xantophores win the competition everywhere. As its value increases, melanophores start to fill more and more the space outside the forbidden regions, which first leads to the formation of black dots, then black stripes and eventually to the essentially black skin with yellow dots.

It is worth noting that the model generates stationary patterns with a wavelength of only a few cells. In many instances, the stochasticity of the different processes in RD systems leads to levels of noise such that pattern formation is compromised at small scales. In this respect, the nonlocal interactions between immobile agents seems to give rise to structures that are especially robust. This is in agreement with the experiments, which show that patterns can be generated in zebrafish whose half wavelength is of the order of 10 cells.

In addition, we also assessed qualitatively the effect of having a pre-pattern on the development of the striped pattern. It is known that the initial stage of the pattern in the trunk of the wild-type zebrafish consists of a single band of iridophores, which inhibits the growth of melanophores on top of them^15^. We simulated this by defining a region (the shaded horizontal bands in Fig. 1) where melanophores cannot grow, or in other words where processes (3) and (8) are completely inhibited. We observed that the presence of such a pre-pattern leads to a selection of the final orientation of the stripes, which often tend to align with the band of iridophores. However, the pre-pattern does not seem to affect the intrinsic wavelength of the pattern itself. More particularly, the final orientation of the stripes in the absence of initial iridophores (Fig. 1a) is such that the system accommodates optimally an integer number of stripes with a wavelength fixed by the choice of parameter values. The presence of a horizontal band (Fig. 1b,c) where the growth of melanophores is inhibited acts as a preferential region for the growth of a xantophores stripe, which in turn directs the evolution of the pattern horizontally. This effect is independent of the size of the pre-pattern: even a narrow band of iridophores can trigger the above mentioned orientation process.

### Analytical results

Simulations reveal the existence of stationary patterns with an intrinsic wavelength, but are these structures the consequence of a Turing bifurcation? To answer that fundamental question, one can perform a linear stability analysis of the equations (14) and (15) in their full generality. This analysis shows that the system can have up to three homogeneous steady states, one of which can undergo a Turing bifurcation. The explicit forms of these solutions are, however, complicated functions of the control parameters. To highlight the origin of the bifurcation, we will rather focus on the simple, yet representative limit of the model with which we performed the simulations (*d*_X_=*d*_M_=*b*_M_=0, *s*_X_=*s*_M_=*s*).

Under these approximations, the system still has three homogeneous steady states


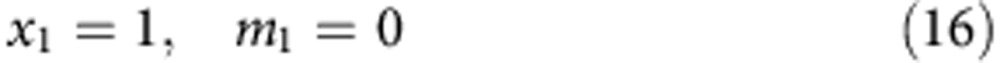



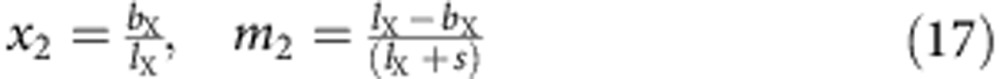



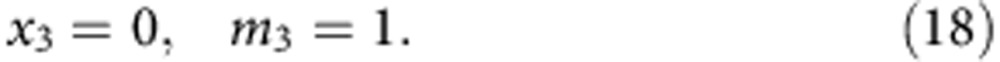


The first and the third states represent a complete saturation of the skin by xantophores or by melanophores, respectively. They appear only because we neglected the natural death of both species. On top of being unrealistic for long times, it can be proven that they cannot undergo a Turing bifurcation. The second state corresponds to a skin containing both types of chromatophores. It has acceptable values only if *l*_X_≥*b*_X_, in other words only if the xantophore-induced birth of melanophores is as efficient as (or more efficient than) the natural birth of xantophores.

This latter state can undergo a Turing bifurcation, which can be expressed in terms of a critical long-range interaction distance *h*_T_


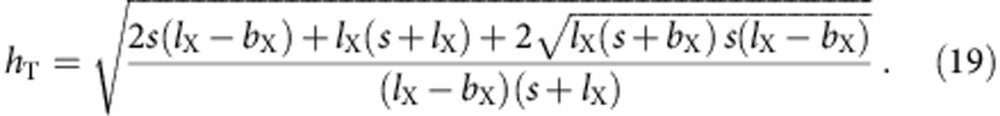


**Figure 3:**
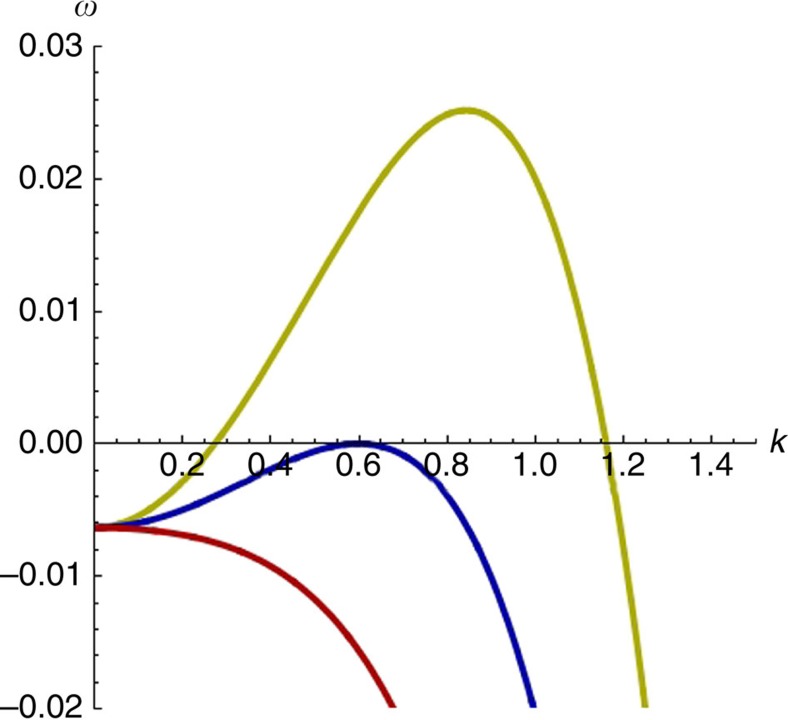
Dispersion relations. Dispersion relations for three representative points before (red, *h*=7), at (blue, *h*=8) and after (yellow, *h*=9) the Turing bifurcation. For the three curves *d*_X_=*d*_M_=*b*_M_=0, *s*=1, *l*_X_=1.0193 and *a*=1.

The corresponding curve is depicted in Fig. 2. Patterns will appear for interaction distances that are larger or equal to this value. Moreover, at the bifurcation point, the most unstable mode of wavenumber *k*_T_ corresponds to a well-defined wavelength (see also Fig. 3), given by


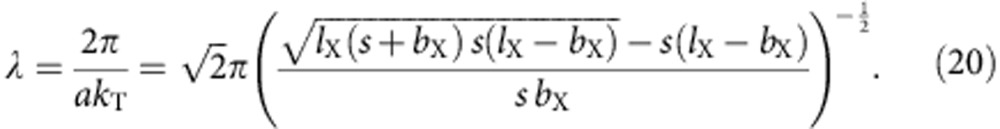


These expressions fit well with the outcomes of the simulations. In particular, the analysis predicts that at the criticality, *λ*≈2*h*_T_ for a wide range of parameters. We can thus conclude that the patterns observed in the simulations of the model are indeed the consequence of a Turing bifurcation, despite the absence of motion of any kind. This is made possible by the presence of the effective cross-diffusion-like terms that we mentioned earlier, which are the mathematical translation of the existence of nonlocal interactions.

## Discussion

On the basis of the above results, we can answer some of the questions that we outlined at the beginning.

We showed that the combination of short-range and long-range feedbacks in the Kondo mechanism can give rise to dynamical models undergoing a Turing bifurcation in the absence of cellular transport. The bifurcation occurs because the combination of the different feedbacks, here between melanophores and xantophores, generates a dynamics of the ‘short-range activation and long-range inhibition' type. As a consequence, patterns with an intrinsic wavelength arise that can rightfully be called Turing patterns. The model we used to reach this conclusion is the simplest possible implementation of the mechanism put forth to explain the emergence of patterns on the skin of zebrafish. It is unlikely that this minimal model will reproduce all the experimental observations, but we can reasonably expect that including additional ingredients, such as the role of iridophores or limited cell motion, will allow for a finer modelling of the experiments, without threatening the above general conclusion.

The mechanism by which the patterns emerge in the zebrafish is hence different from the proposal by Alan Turing. The proposed feedbacks consist in a combination of nonlocal biological interactions that affect the growth rates of the cells differently, depending on their surrounding. We propose to call this mechanism *differential growth*. The key element to the emergence of the pattern is that, although the single cells are immobile, differential growth induces an effective redistribution of cell populations in space. It would be of interest to assess whether similar feedbacks are at the heart of pattern formation in other instances of morphogenesis, so as to clarify the degree of universality of this mechanism. Moreover, efforts should be made to elucidate the exact physico-chemical nature of the cell-to-cell coupling to understand how the skin patterns enter the general framework of dissipative structures.

## Methods

### Simulation algorithm

To simulate the stochastic processes standing for the different steps of the proposed regulatory scheme, we used Kinetic MC simulations based on the following algorithm:

1. The substrate is modelled as a square lattice with periodic boundary conditions, composed of *N*_0_ nodes having a coordinancy of 4 (that is, there are four first neighbours). Each of these nodes can be occupied by a xantophore (state X), by a melanophore (state M) or by none of these chromatophores (state S). An initial configuration of the lattice is chosen. In the simulations presented in this work, the lattice was initially uniformly covered in S.
2. A probability *P*_*k*_ is associated with each of the elementary steps of the scheme. It is calculated as the rate constant of the process divided by the sum of all the rate constants.
3. One site of the lattice, say position *i*, is chosen at random and one of the processes is selected (with the appropriate probability). If the spatial configuration of the cells is in accordance with the chosen process, the composition of the lattice is changed accordingly. To be more precise:
4. Whatever the process, and whatever the result of the corresponding attempt, time is advanced by 1/*N*_0_ and the algorithm goes back to 3.

In some of the simulations presented in the main text, we simulated the presence of a band of iridophores, which inhibit the growth of melanophores on top of them. We implemented this feature by defining a region of space in the form of a horizontal line, where the birth of melanophores (either natural or induced by the long-range interaction with xantophores) is not possible. We keep this condition on for the whole duration of the simulation.

**Interactive JavaScript simulator:** This paper is accompanied by Supplementary Software. The Supplementary Information contains a freely accessible interactive JavaScript software, which allows the interested reader to perform customised Monte Carlo simulations of the presented model.

## Author contributions

D.B. and Y.D.D. designed and developed the model, performed simulations, analysed the results and wrote the manuscript.

## Additional information

**How to cite this article:** Bullara, D. and De Decker, Y. Pigment cell movement is not required for generation of Turing patterns in zebrafish skin. *Nat. Commun*. 6:6971 doi: 10.1038/ncomms7971 (2015).

## Supplementary Material

Supplementary Software Supplementary Software
