# Supplementary material for: Pigment cell movement is not required for generation of Turing patterns in zebrafish skin
Source: Nat Commun. 2015 May 11;6:6971. doi: 10.1038/ncomms7971 (PMC4432648; doi:10.1038/ncomms7971)
Supplement: Supplementary Software [file ncomms7971-s1.zip › data/js/jquery-ui-1.11.2.custom/index.html]

jQuery UI Example Page


# Welcome to jQuery UI!

This page demonstrates the widgets and theme you selected in Download Builder. Please make sure you are using them with a compatible jQuery version.

# YOUR COMPONENTS:

## Button

A button element


Choice 1
Choice 2
Choice 3


## Dialog

Open Dialog

## Overlay and Shadow Classes *(not currently used in UI widgets)*

Lorem ipsum dolor sit amet, Nulla nec tortor. Donec id elit quis purus consectetur consequat.

Nam congue semper tellus. Sed erat dolor, dapibus sit amet, venenatis ornare, ultrices ut, nisi. Aliquam ante. Suspendisse scelerisque dui nec velit. Duis augue augue, gravida euismod, vulputate ac, facilisis id, sem. Morbi in orci.

Nulla purus lacus, pulvinar vel, malesuada ac, mattis nec, quam. Nam molestie scelerisque quam. Nullam feugiat cursus lacus.orem ipsum dolor sit amet, consectetur adipiscing elit. Donec libero risus, commodo vitae, pharetra mollis, posuere eu, pede. Nulla nec tortor. Donec id elit quis purus consectetur consequat.

Nam congue semper tellus. Sed erat dolor, dapibus sit amet, venenatis ornare, ultrices ut, nisi. Aliquam ante. Suspendisse scelerisque dui nec velit. Duis augue augue, gravida euismod, vulputate ac, facilisis id, sem. Morbi in orci. Nulla purus lacus, pulvinar vel, malesuada ac, mattis nec, quam. Nam molestie scelerisque quam.

Nullam feugiat cursus lacus.orem ipsum dolor sit amet, consectetur adipiscing elit. Donec libero risus, commodo vitae, pharetra mollis, posuere eu, pede. Nulla nec tortor. Donec id elit quis purus consectetur consequat. Nam congue semper tellus. Sed erat dolor, dapibus sit amet, venenatis ornare, ultrices ut, nisi. Aliquam ante.

Suspendisse scelerisque dui nec velit. Duis augue augue, gravida euismod, vulputate ac, facilisis id, sem. Morbi in orci. Nulla purus lacus, pulvinar vel, malesuada ac, mattis nec, quam. Nam molestie scelerisque quam. Nullam feugiat cursus lacus.orem ipsum dolor sit amet, consectetur adipiscing elit. Donec libero risus, commodo vitae, pharetra mollis, posuere eu, pede. Nulla nec tortor. Donec id elit quis purus consectetur consequat. Nam congue semper tellus. Sed erat dolor, dapibus sit amet, venenatis ornare, ultrices ut, nisi.

Lorem ipsum dolor sit amet, consectetur adipisicing elit, sed do eiusmod tempor incididunt ut labore et dolore magna aliqua. Ut enim ad minim veniam, quis nostrud exercitation ullamco laboris nisi ut aliquip ex ea commodo consequat.

Lorem ipsum dolor sit amet, consectetur adipisicing elit, sed do eiusmod tempor incididunt ut labore et dolore magna aliqua. Ut enim ad minim veniam, quis nostrud exercitation ullamco laboris nisi ut aliquip ex ea commodo consequat.

## Framework Icons (content color preview)


## Highlight / Error

**Hey!** Sample ui-state-highlight style.

  

**Alert:** Sample ui-state-error style.
