# Supplementary material for: Pigment cell movement is not required for generation of Turing patterns in zebrafish skin
Source: Nat Commun. 2015 May 11;6:6971. doi: 10.1038/ncomms7971 (PMC4432648; doi:10.1038/ncomms7971)
Supplement: Supplementary Software [file ncomms7971-s1.zip › index.html]

App M


Start timeStop time  
Re-initialize
Randomize  
  

## PRESETS

---

Black spots
Stripes
Yellow spots
Random birth and death
Random birth + short-range competition  


---

  
Set simulation parameters

- Si

  bX=

  1

  Xi
- Si

  bM=

  1

  Mi
- Xi

  dX=

  1

  Si
- Mi

  dM=

  1

  Si
- Mi±1 + Xi

  sM=

  1

  Mi±1 + Si
- Xi±1 + Mi

  sX=

  1

  Xi±1 + Si
- Xi±h + Si

  lX=

  1

  Xi±h + Mi

- h=

  15

Linear size of the lattice (in number of cells)

Width of the iridophore band (in number of cells)

Linear size of each lattice node (in number of pixels)

Number of time steps per screen refresh

Minimum delay per screen refresh (in milliseconds)
