# Supplementary figures and images for: Pigment cell movement is not required for generation of Turing patterns in zebrafish skin
Source: Nat Commun. 2015 May 11;6:6971. doi: 10.1038/ncomms7971 (PMC4432648; doi:10.1038/ncomms7971)

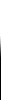

Supplement: Supplementary Software [file ncomms7971-s1.zip › data/js/jquery-ui-1.11.2.custom/images/ui-bg_highlight-soft_100_eeeeee_1x100.png]

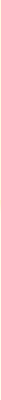

Supplement: Supplementary Software [file ncomms7971-s1.zip › data/js/jquery-ui-1.11.2.custom/images/ui-bg_glass_100_fdf5ce_1x400.png]

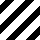

Supplement: Supplementary Software [file ncomms7971-s1.zip › data/js/jquery-ui-1.11.2.custom/images/ui-bg_diagonals-thick_20_666666_40x40.png]

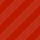

Supplement: Supplementary Software [file ncomms7971-s1.zip › data/js/jquery-ui-1.11.2.custom/images/ui-bg_diagonals-thick_18_b81900_40x40.png]

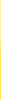

Supplement: Supplementary Software [file ncomms7971-s1.zip › data/js/jquery-ui-1.11.2.custom/images/ui-bg_highlight-soft_75_ffe45c_1x100.png]

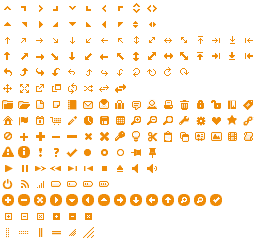

Supplement: Supplementary Software [file ncomms7971-s1.zip › data/js/jquery-ui-1.11.2.custom/images/ui-icons_ef8c08_256x240.png]

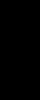

Supplement: Supplementary Software [file ncomms7971-s1.zip › data/js/jquery-ui-1.11.2.custom/images/ui-bg_flat_10_000000_40x100.png]

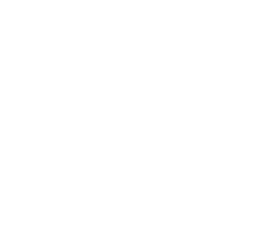

Supplement: Supplementary Software [file ncomms7971-s1.zip › data/js/jquery-ui-1.11.2.custom/images/ui-icons_ffffff_256x240.png]

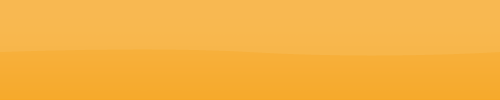

Supplement: Supplementary Software [file ncomms7971-s1.zip › data/js/jquery-ui-1.11.2.custom/images/ui-bg_gloss-wave_35_f6a828_500x100.png]

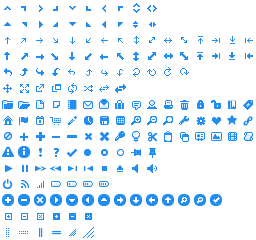

Supplement: Supplementary Software [file ncomms7971-s1.zip › data/js/jquery-ui-1.11.2.custom/images/ui-icons_228ef1_256x240.png]

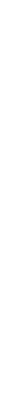

Supplement: Supplementary Software [file ncomms7971-s1.zip › data/js/jquery-ui-1.11.2.custom/images/ui-bg_glass_65_ffffff_1x400.png]

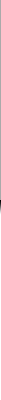

Supplement: Supplementary Software [file ncomms7971-s1.zip › data/js/jquery-ui-1.11.2.custom/images/ui-bg_glass_100_f6f6f6_1x400.png]

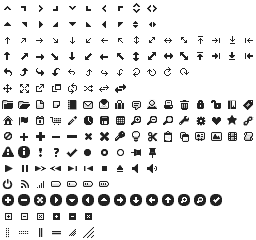

Supplement: Supplementary Software [file ncomms7971-s1.zip › data/js/jquery-ui-1.11.2.custom/images/ui-icons_222222_256x240.png]

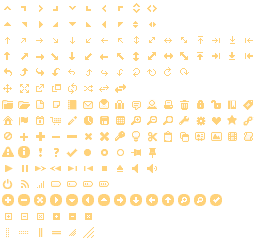

Supplement: Supplementary Software [file ncomms7971-s1.zip › data/js/jquery-ui-1.11.2.custom/images/ui-icons_ffd27a_256x240.png]
